# A Rare Case of Inguinal Hernia of a Ureter Belonging to a Duplex Kidney

**DOI:** 10.1155/2023/1285212

**Published:** 2023-06-27

**Authors:** Mohadese Dashtkoohi, Azade Haghiri, Mohammad Sadeq Najafi

**Affiliations:** School of Medicine, Tehran University of Medical Sciences, Tehran, Iran

## Abstract

**Introduction:**

Inguinal herniation of the ureter is a rare entity that occurs either as a complication of renal transplantation or spontaneously. Patients may suffer from obstructive uropathy or groin pain due to the unusual ectopic course of the ureter. This case report highlights the importance of recognizing a ureteroinguinal hernia.

**Methods:**

In this case report, we present a 75-year-old man with a surgical history of a right inguinal hernia repair who was referred to our center with burning left inguinal pain that persisted for two weeks. The patient's history and physical examination were consistent with an inguinal hernia. The suspected indirect inguinal hernia was found on preoperative imaging to be a tubular structure distinct from the intestine or adjacent organs. An open exploration of the inguinal canal was performed to prevent further hernia development.

**Results:**

The unusual structure in the inguinal canal turned out to be an ectopic ureter originating from the left upper pole moiety of the left duplex kidney (i.e., with duplicated ureters) and containing concentrated urine, as confirmed on a postoperative computerized tomography urogram.

**Conclusion:**

It is crucial to perform a thorough clinical examination and utilize adequate imaging modalities before surgical procedures when encountering unidentified structures.

## 1. Introduction

Inguinal hernias affect up to 40% of men and 5% of women during their lifetime and account for about one-third of all hernias [1, 2]. Inguinal herniorrhaphy is a very common procedure worldwide [3]. Nevertheless, inguinal hernia of the ureter is a rare event, occurring either as a complication of renal transplantation or spontaneously [4, 5]. Ureteroinguinal hernia may lead to obstructive uropathy [6, 7]; however, as in the present case, the patient may have relatively nonspecific symptoms. This case report describes a patient who was initially diagnosed with a left inguinal hernia but was ultimately discovered to have an inguinal hernia of the ureter, with no evidence of obstructive uropathy. In most patients with normal laboratory values and no urinary complaints, ureteroinguinal herniation is overlooked as a potential diagnosis before surgery.

## 2. Case Presentation

A 75-year-old non-smoker man with a surgical history of a right inguinal hernia repair was referred to our center with burning pain in the left inguinal region that persisted for two weeks. He also complained of a left inguinal mass, which was later examined by ultrasound (US). Physical examination revealed a reducible left inguinal mass suggestive of a hernia but with no evidence of strangulation. Furthermore, testicular and abdominopelvic examinations were normal. The first US examination did not reveal a hernia sac but did demonstrate a cystic fluid-filled lesion, so the patient underwent the second US for confirmation. During this time, the patient's inguinal pain was relieved by intramuscular ketorolac 30 mg twice a day. The second US examination revealed a cystic lesion in the inguinal canal, which was believed to be a tortuous vas deferens. The radiologist recommended a pelvic Magnetic Resonance Imaging (MRI) scan to better characterize this unusual structure. MRI showed a large tortuous cystic lesion on the left side of the pelvic cavity, posterior and superior to the urinary bladder, extending to the seminal vesicle, and suggestive of a possible seminal vesicle cyst versus a vas deferens cyst. Since the diagnosis could not be confirmed by MRI, a urologist was consulted to evaluate the hernia and the suspected seminal vesicle cyst. The urologist recommended surgical exploration of the area.

**Figure 1:**
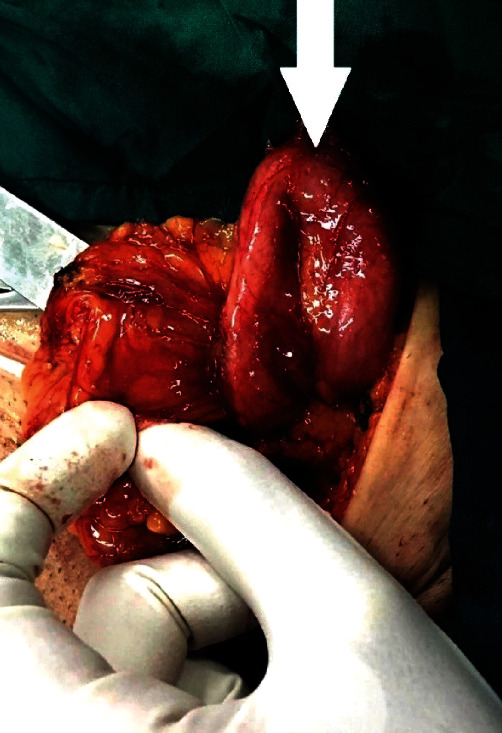
Surgical exploration shows a rare ectopic ureter (U-shaped structure) in the inguinal canal as shown by the arrow.

**Figure 2:**
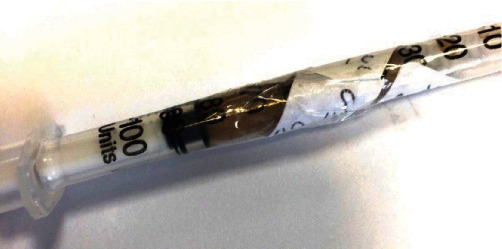
An insulin syringe containing aspirated fluid from the U-shaped structure.

The patient underwent surgical exploration of the left inguinal canal. After prepping and draping, a classic incision for hernia repair was made under spinal anesthesia. The skin, fascia, and inguinal canal were opened, then the spermatic cord was identified and kept aside from the field to avoid its damage while exploring the area. Interestingly, no hernia sac was found. A tubular U-shaped structure distinct from the intestine or adjacent organs was identified in the inguinal canal (Figure 1). The U-shaped structure, which originated in the retroperitoneum, passed through the internal ring and was positioned alongside the cord but never entered the scrotum. The fluid inside the U-shaped structure was then aspirated, and fine-needle aspiration of the tissue was performed in consultation with a urologist for later examination. The contents of the aspiration were a yellowish-brown turbid fluid, shown in Figure 2. A detailed exploration of the retroperitoneum was performed to characterize the unknown structure as much as possible. Since we were unable to originate and identify the structure, suspicions were raised about a left-side urinary system malformation. We decided to perform an open repair of the surrounding tissue of the inguinal canal to prevent further development of the hernia. The U-shaped structure was pushed back into the retroperitoneum and otherwise left untouched (in consultation with the urologist). Finally, the viscera were retracted, then the inguinal canal, Scarpa's fascia, and skin were sutured sequentially. For postoperative pain management, intravenous acetaminophen (1 g every 8 hours) and intramuscular pethidine (50 mg every 6 hours) were prescribed [8].

**Figure 3:**
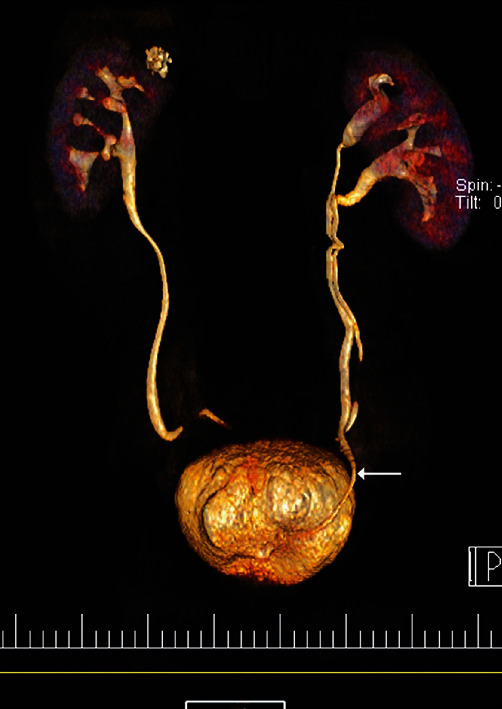
CT urogram showing duplex (i.e., with double ureters) left kidney. The left upper moiety ureter that empties into the bladder at an ectopic site is indicated by the arrow.

The analysis of the fluid was negative for sperm. It was also culture negative. The biopsy specimen revealed normal ureteral histology with stratified transitional epithelium. Based on the pathology findings and the high creatinine (Cr) value of the aspirated fluid from the U-shaped lesion (Cr = 13.1 mg/dL), the patient's urinary system was restudied with a computerized tomography (CT) urogram. CT urogram demonstrated a duplicated left collecting system (Figure 3). The right ureter orthotopically emptied into the urinary bladder, whereas the left ureter took ectopic courses. The left lower pole moiety ureter was inserted into the urinary bladder orthotopically, as shown in Figure 3, whereas the left upper pole moiety ureter was inserted more inferio-medially into the inguinal canal and caused dilatation of the seminal vesicle.

The patient was referred to a urologist for further evaluation. The urologist believed in the congenital nature of this malformation and that age-related weakness of the surrounding tissue resulted in inadequate support of the ectopic structure, causing it to descend into the inguinal canal. Because of the high risk of surgery at the age of 75 years and the fact that the patient had not suffered renal failure during most of his life (Cr = 0.9 mg/dL, estimated glomerular filtration rate ((eGFR) = 89), he was treated only with an appropriate analgesic (paracetamol 500 mg according to the need of the patient, maximum daily dose = 4 g). At follow-up 6 months later, the patient no longer complained of lower urinary tract pain or symptoms.

**Figure 4:**
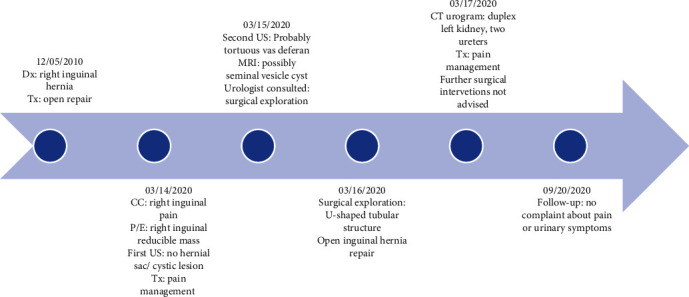
Summary of the events is depicted in the timeline. Dates are written in MM/DD/YYYY format. Dx: diagnosis, Tx: treatment, CC: chief complaint, P/E: physical examination, US: ultrasound examination.

The summary of the events is depicted in the timeline (see Figure 4).

### 2.1. Ethical Approval

The patient was treated under the latest version of the Declaration of Helsinki of the World Medical Association [9], and written informed consent was obtained before each procedure.

## 3. Discussion

Inguinal hernias account for nearly 75% of all hernias, 50% of which occur indirectly, often on the right side (7 : 1: ratio of men to women) [1]. Ureteral hernia a rarely observed phenomenon with less than 150 cases reported worldwide [10], can occur inguinally, femorally, thoracically, or para iliac [11, 12]. Ureteroinguinal hernia is an infrequent condition [5] that occurs more often in association with inguinal hernia compared with other types of hernia [13, 14], and is usually misdiagnosed preoperatively. It also remains challenging to clinically differentiate from an inguinal hernia, hydrocele, or seminal vesicle cyst, since the ectopic ureteral insertion can cause seminal vesicle dilation [15–17].

Based on the accompanying structures, a ureteral hernia is classified as paraperitoneal or extraperitoneal. The former accounts for 80% of cases [18]. Renal transplantation remains the major risk factor for the paraperitoneal type, whereas congenital malformation of the ureter is typically responsible for the extraperitoneal type [19, 20]. Other risk factors include male gender, age >50 years, obesity, and collagen synthesis deficiencies [21]. Our patient was a man over 50 years with a history of inguinal hernia as a risk factor.

The diagnosis of ureteroinguinal hernia is often not made preoperatively [16] and relies on imaging techniques, such as MRI [22] and CT urogram [23]. The rationale behind the MRI examination of the inguinal hernia before surgery in the present case was the high sensitivity (>90%) and specificity (>90%) of MRI in diagnosing inguinal hernia, especially occult hernia [24].

Surgical treatment of ureteroinguinal hernias remains controversial [14, 25]. Some surgeons advise open or laparoscopic herniorrhaphy to avoid strangulation and obstructive uropathy, whereas others recommend no hernia repair to avoid injury to the ureter. A ureteral stent can be inserted preoperatively to protect the ureter during herniorrhaphy [18, 26, 27]. In our case, we preferred not to dissect the suspicious structure before further examination, and herniation of the ureter was diagnosed postoperatively.

In this study, we present an extremely rare case of an ectopic ureter with no urinary symptoms that herniated from an extraperitoneal origin into the inguinal canal. The final diagnosis was made with the help of the CT urogram, which visualized the ureteral tract with extraordinary precision and showed that the ureter from the upper pole moiety of the duplicated collecting system of the left kidney passed through the inguinal canal, mimicking an inguinal hernia. The course of duplicated left ureters was consistent with the Weigert-Meyer law [28].

The main limitation of this case report was that the urologist was consulted by telephone and was absent during the operation. The unavailability of intraoperative imaging was also an important limitation of this study.

Because ureteroinguinal hernia is quite rare, the definitive diagnosis is usually based on imaging and surgical exploration. Imaging methods, such as CT urogram and MRI, are preferred because of their higher diagnostic value. Therefore, we recommend a complete examination of the patient's urinary tract before surgery if urinary symptoms are present or if there is reasonable doubt about congenital abnormalities. This would help to avoid blind intervention and irreparable complications.

## Data Availability

Data supporting this research article are available from the corresponding author or first author upon reasonable request.
